# Duodenocaval Fistula Caused by Post-Bulbar Duodenal Ulcer: A Case Report

**DOI:** 10.70352/scrj.cr.25-0153

**Published:** 2025-07-09

**Authors:** Hirotaka Ishido, Hidehiro Tajima, Soya Meguro, Musashi Takada, Teppei Tatsuoka, Keishi Kawasaki, Takashi Okuyama, Hideyuki Yoshitomi

**Affiliations:** Department of Surgery, Dokkyo Medical University Saitama Medical Center, Koshigaya, Saitama, Japan

**Keywords:** duodenocaval fistula, post-bulbar duodenal ulcer, invasive *Candida* infection

## Abstract

**INTRODUCTION:**

Duodenocaval fistula (DCF) is a rare, life-threatening disease with a mortality rate of approximately 40%. There are several types of etiologies for DCF. Among these, duodenal ulcers are the most dangerous, with a mortality rate of 63.6%. This report describes a case of DCF caused by a duodenal ulcer that was successfully diagnosed and treated, and discusses why duodenal ulcer-induced DCF results in severe outcomes, with a review of the literature.

**CASE PRESENTATION:**

A 64-year-old male patient was transferred to our hospital for surgical treatment of DCF. An emergency laparotomy was performed. We opened the abscess cavity, repaired the perforation site and inferior vena cava, and performed gastrojejunostomy. After the operation, anastomotic leakage was suspected. Although we treated him with antibiotics and antifungal drugs, his condition did not improve, and he experienced elevated total bilirubin and inflammation. Therefore, we decided to perform a reoperation on postoperative day (POD) 30, including cholecystectomy, insertion of a C-tube into the common bile duct for biliary drainage, enterostomy, and peritoneal irrigation and drainage. After that, the patient continued antibiotic and antifungal therapy, with periodic drain flushing and replacement. His condition slowly improved, and he was transferred to another facility for rehabilitation on POD 158.

**CONCLUSIONS:**

DCF caused by duodenal ulcers has a higher mortality rate than that caused by other etiologies. Therefore, initiating broad-spectrum antibiotics and antifungal therapy early may be beneficial. Also, it is reasonable to perform simple, rapid, and minimally invasive surgery in an emergency setting. However, even in emergency situations, duodenal decompression, cholecystectomy, and biliary drainage should be performed to prevent anastomotic leakage and biliary complications.

## Abbreviations

DCF
: duodenocaval fistula
ICI
: invasive *Candida* infection
IVC
: inferior vena cava
PBDU
: post-bulbar duodenal ulcer

## INTRODUCTION

DCF is a rare, life-threatening disease with a mortality rate of approximately 40%.^1)^ Its etiologies include migrating cava filters, abdominal trauma, foreign bodies, peptic duodenal ulcers, retroperitoneal tumor resection with radiotherapy, and bevacizumab treatment. Among these, duodenal ulcers are the most dangerous, with a mortality rate of 63.6%.^2)^ Traditionally, DCF is diagnosed as gastrointestinal bleeding or septic syndrome. Although rapid diagnosis and treatment are essential to save patients with DCF, most patients die before they can undergo surgery.^2)^

Herein, we report a successfully diagnosed and treated case of DCF caused by a duodenal ulcer and discuss why duodenal ulcer-induced DCF results in severe outcomes, with a review of the literature.

## CASE PRESENTATION

**Fig. 1:**
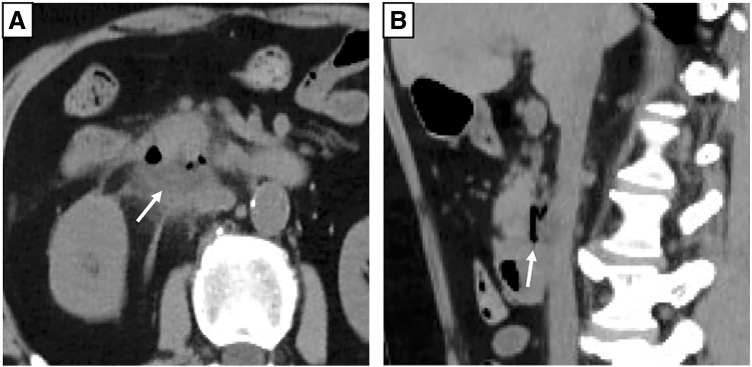
CT scan from previous hospitalization. A white arrow in both axial (**A**) and sagittal (**B**) CT scans indicates dorsal duodenal purulent effusion and internal air. CT, computed tomography

**Fig. 2:**
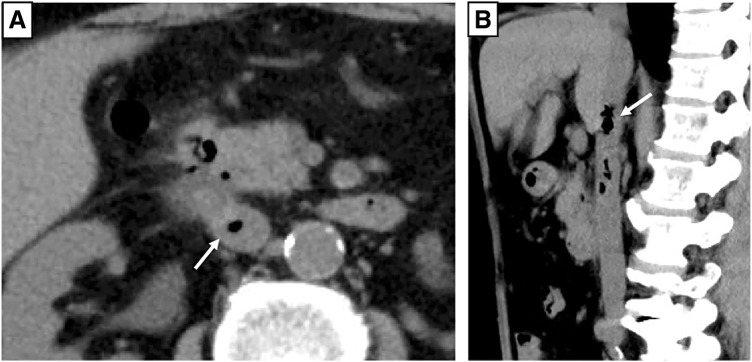
CT scan when the patient complained of abdominal pain. A white arrow in both axial (**A**) and sagittal (**B**) CT scans indicates an air bubble in the inferior vena cava. CT, computed tomography

A 64-year-old male patient was rushed to another hospital due to immobility. He exhibited hyperpnea and epigastric tenderness but no rebound tenderness. His medical history included hypertension, duodenal bulb ulcers, and *Helicobacter pylori* infection. He was taking acetaminophen, loxoprofen sodium hydrate, trichlormethiazide, and nifedipine. Enhanced CT revealed abscess formation and free air behind the descending part of the duodenum (**Fig. 1**). Routine blood tests revealed elevated inflammatory markers (white blood cell count: 33730/μL; C-reactive protein [CRP]: 33.95 mg/dL), anemia (hemoglobin: 7.1 g/dL), and renal dysfunction (blood urea nitrogen [BUN]: 77.7 mg/dL; creatinine: 2.30 mg/dL). The patient was diagnosed with peptic ulcer perforation and acute renal failure. Blood culture was positive for *Candida albicans* (β-D-glucan: 1039.0 pg/mL). Therefore, he was admitted to the previous hospital, received blood transfusions, and was treated with antibiotics and antifungal drugs for 1 week; however, his condition did not improve. The patient complained of abdominal pain. Repeat CT revealed air bubbles in the IVC, suggesting DCF (**Fig. 2**). He was then transferred to our hospital (Dokkyo Medical University Saitama Medical Center, Saitama, Japan) for surgery. Physical examination revealed tenderness from the epigastric area to the right quadrant, no muscular defense, and no rebound tenderness. Blood tests showed slight improvement in renal function (BUN: 47 mg/dL; creatinine: 1.74 mg/dL); however, inflammation remained high (white blood cell count: 23700/μL; CRP: 24.03 mg/dL), and the patient was anemic (hemoglobin: 7.4 g/dL) and developed disseminated intravascular coagulation (platelet count: 132000; prothrombin time/normal prothrombin ratio [PT/PTNL]: 1.29; fibrin/fibrinogen degradation products [FDP]: 396.7 μg/mL).

### Surgical treatment

**Fig. 3:**
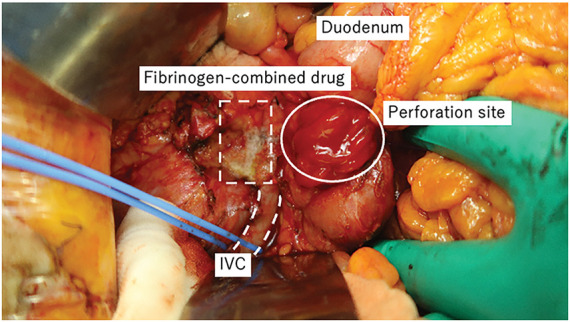
Photos at the time of surgery. The white circle indicates the site of perforation of the duodenum. The white dotted square indicates the site where fibrinogen-combined drug was used to stop bleeding from the IVC. The white double-dotted line indicates the IVC taped with blue tape. IVC, inferior vena cava

An emergency laparotomy with a vertical midline incision was performed. There were no obvious adhesions in the abdominal cavity. The ascites was serous rather than purulent. Then, Kocher’s maneuver was performed. The abscess cavity was opened, revealing bile-like contents. The culture from the ascites was negative, but the culture from the abscess was positive for *C. albicans*. The duodenal perforation site was edematous, measuring approximately 3 cm. During dissection around the abscess cavity, bleeding was observed from the right lateral wall of the IVC, likely causing its perforation. Astriction failed to control the bleeding; therefore, we consulted cardiovascular surgeons for suture control. They taped the IVC above and below the site to block the bleeding and tried to suture; however, due to high blood flow and a fragile, inflamed IVC wall, suture closure was deemed difficult. Therefore, a fibrinogen-combined drug was applied to stop the bleeding (**Fig. 3**). The duodenal perforation site was closed using an Albert–Lembert anastomosis with an omental patch. To prevent suture stricture, we performed gastrojejunostomy with a partition and placed a drainage tube at the foramen of Winslow. The ascites was serous and the abscess cavity was localized, so we did not place an additional drainage tube (operation time: 274 min; bleeding: 1810 mL; blood transfusion: Red Blood cells [RBCs] 10 units, Fresh Frozen Plasma [FFP] 4 units).

### Postoperative course

**Fig. 4:**
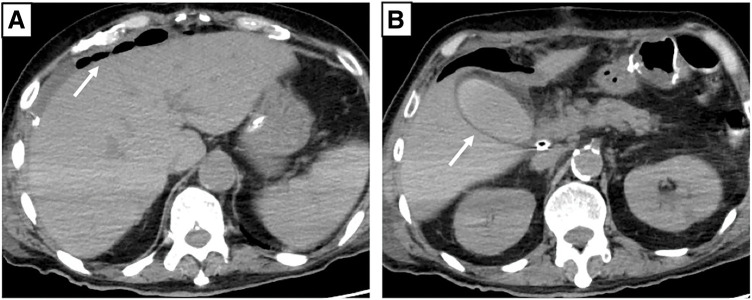
CT scan before reoperation. The CT scan shows no intrahepatic bile duct dilatation, and a white arrow indicates increased free air around the liver (**A**). A white arrow indicates thickening of the gallbladder wall (**B**). CT, computed tomography

**Fig. 5:**
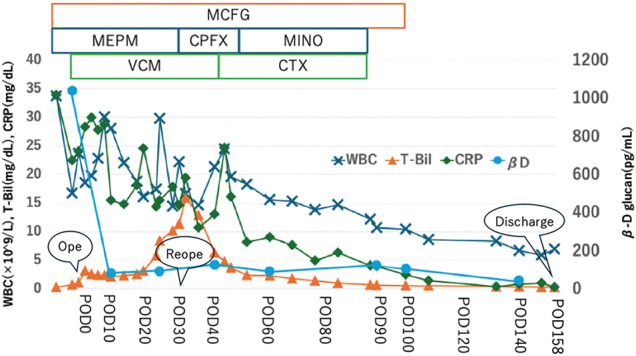
Clinical course. Antibiotics were changed each time based on each culture, and the antifungal agent was continued until βD levels decreased. βD, β-D-glucan; CPFX, Ciprofloxacin; CRP, C-reactive protein; CTX, Cefotaxime; MCFG, Micafungin; MEPM, Meropenem; MINO, Minocycline; POD, postoperative day; T-Bil, total bilirubin; VCM, Vancomycin; WBC, white blood cell count

Biliary ascites was observed through the drain on POD 4, raising suspicion of anastomotic leakage. The drain was flushed daily with normal saline and replaced weekly, along with intravenous antibiotics and antifungal drugs. However, the patient’s condition did not improve, and total bilirubin and inflammation increased (aspartate aminotransferase: 54 U/L; alanine aminotransferase: 40 U/L; alkaline phosphatase: 218 U/L; gamma-glutamyl transpeptidase: 144 U/L; total bilirubin: 10.87 mg/dL; white blood cell count: 16300/μL; CRP: 21.55 mg/dL). Repeat CT showed no intrahepatic bile duct dilatation or residual abscess; however, gallbladder wall thickening, free air around the liver, and increased fatty tissue around the gallbladder were observed (**Fig. 4**). Cholecystitis was suspected, but we thought that the condition was attributable to elevated intrabiliary pressure due to duodenal stenosis distal to the papilla of Vater. We considered endoscopic intervention; however, clinical improvement was not expected with this approach. Although CT did not clearly show residual abscess formation, intra-abdominal infection could not be ruled out. Therefore, reoperation was performed on POD 30, including cholecystectomy, insertion of a C-tube into the common bile duct for biliary drainage, enterostomy, and peritoneal irrigation and drainage. In fact, a residual abscess cavity was observed at the caudoventral side of the IVC, but the IVC perforation site was completely repaired. Also, we did not find anastomotic leakage, and it was considered to have been successfully managed conservatively. However, it was thought that a stricture developed during the healing process. Drains were placed in the right subphrenic space, abscess cavity, and subhepatic space. The patient continued antibiotic and antifungal therapy, with periodic drain flushing and replacement. Bilirubinemia and inflammation gradually improved, and blood culture confirmed the absence of fungemia (β-D-glucan: 44.1 pg/mL). After the removal of all drainage tubes, the patient was unable to resume oral intake and remained dependent on enteral nutrition via enterostomy. He was transferred to another facility for rehabilitation on POD 158 (**Fig. 5**).

## DISCUSSION

DCF is a critical condition with a poor prognosis, even when diagnosed promptly. DCF caused by duodenal ulcers has a higher mortality rate than that caused by other etiologies.^2)^ Several factors contribute to this high mortality.

First, only large ulcers lead to DCF (range: 1.5–9 cm),^2)^ increasing the risk of IVC bleeding. Additionally, the large fistula size may allow bacteria to enter the bloodstream, increasing susceptibility to sepsis. Second, given the anatomy of the hepatoduodenal ligament and pancreas, DCF is associated with PBDUs. Large PBDUs cause significant leakage of gastric acid, bile, and pancreatic juice, which can impair wound healing and promote rebleeding.^3)^ Generally, mixed bile and pancreatic juice activate digestive enzymes, exacerbating abscess formation and sepsis. PBDU is more common in males, though factors such as age and medical history vary from region to region and are reported; therefore, its etiology remains unsolved.^4)^ Matsuhashi et al. reported that PBDU is unrelated to *Helicobacter pylori* infection, nonsteroidal anti-inflammatory drug use, or psychological stress—common causes of peptic ulcers—but may be linked to gastric hypersecretion.^3)^ Third, inflammation can spread to adjacent organs and blood vessels, leading to further abscess formation and bleeding. Koh et al. reported a recurrent case of DCF due to PBDU with postoperative bleeding.^5)^ Fourth, ICIs occur in 21.7% of patients with perforated peptic ulcers, with a 90-day postoperative mortality rate of 37.8%.^6)^ *C. albicans*, a normal inhabitant of the gastrointestinal tract, may predispose patients with DCF to ICIs. Additionally, *C. albicans* forms biofilms with various bacteria, including *H. pylori*, contributing to treatment resistance.^7,8)^

For these reasons, DCF caused by duodenal ulcers has a poor prognosis. While early diagnosis and surgical treatment are crucial, controlling sepsis and inflammation is equally important. A review of 10 cases of DCF due to duodenal ulcers found that 90% had positive blood cultures, including Gram-negative rods, Gram-positive cocci, and *Candida*, with 55.6% testing positive for *Candida*.^5,9–15)^ Therefore, initiating broad-spectrum antibiotics and antifungal therapy early may be beneficial.

Surgical treatment of DCF includes duodenal and IVC suturing with epiploic or jejunal patches, truncal vagotomy, distal gastrectomy, and/or duodenal exclusion. IVC division or excision is rare, though some cases require pancreaticoduodenectomy with gastrojejunostomy and choledochojejunostomy.^2)^ However, complex surgeries like pancreaticoduodenectomy are often unfeasible in emergencies, particularly in patients with unstable vital signs or massive blood loss. It is reasonable to perform simple, rapid, and minimally invasive surgery. In our case, fibrinogen-combined drugs were effective when IVC suturing was difficult. In fact, Maisano states that a fibrinogen-combined drug is effective when applied to venous vessels that are delicate and difficult to suture. Also, the drug should be applied to the wound so that it covers the area around it by 1–2 cm.^16)^

Even in emergency settings, duodenal decompression, cholecystectomy, and biliary drainage should be performed to prevent anastomotic leakage and biliary complications. These interventions help prevent duodenal juice leakage, which can lead to abscess formation and sepsis. Large PBDUs tend to be edematous due to inflammation, causing digestive juice congestion proximal to the suture site. Posmelov et al. reported duodenal stenosis in 72.7% of patients with PBDU after surgery.^17)^

In this case, we did not consider these interventions during the initial surgery, but this case highlighted their importance. Therefore, duodenal decompression, prophylactic cholecystectomy, and biliary drainage should be considered.

## CONCLUSIONS

We report a rare case of DCF caused by PBDU, a life-threatening condition. To improve survival in DCF caused by duodenal ulcers, early antibiotic and antifungal therapy should be initiated, along with duodenal decompression and cholecystectomy in addition to fistula closure.

## ACKNOWLEDGMENTS

We would like to thank Editage (www.editage.jp) for English language editing.

## DECLARATIONS

### Funding

No funding was received.

### Authors’ contributions

HI, HT, SM, MT, TT, KK, TO, and HY participated in the conception, design, and data acquisition of the study.

TO and HY performed data analysis and interpretation.

HI wrote the manuscript.

HT revised the manuscript.

HT, TO, and HY confirm the authenticity of all the raw data.

All authors have reviewed and approved the final manuscript, and each author agrees to be held accountable for all aspects of the research.

### Availability of data and materials

The data generated in this study may be requested from the corresponding author.

### Ethics approval and consent to participate

Written informed consent was obtained from this patient in accordance with the ethical principles of the 1964 Declaration of Helsinki and its subsequent amendments.

### Consent for publication

Written informed consent was obtained from the patient for the publication of this report and its accompanying images.

### Competing interests

The authors declare that they have no competing interests.
